# Supplementary material for: Forest birds respond to the spatial pattern of exurban development in the Mid-Atlantic region, USA
Source: PeerJ. 2016 May 25;4:e2039. doi: 10.7717/peerj.2039 (PMC4888296; doi:10.7717/peerj.2039)
Supplement: Supplemental Information 1 — Narrow definition refers to the inclusion of scatter isolated pixels (i.e., MSPA class: Islets) in the definition of exurban development, whereas broad definition includes also classes that represented associated roads (i.e., MSPA classes: Bridge, Branch, and Loop). [file peerj-04-2039-s002.docx]

Appendix 1: Landscape composition and configuration surrounding selected Breading Bird Survey stops (n = 125) at 400m- and 1 km-radius buffer (mean ± sd) for 1986, 1993, 2000, and 2009. Narrow definition refers to the inclusion of scatter isolated pixels (i.e., MSPA class: Islets) in the definition of exurban development, whereas broad definition includes also classes that represented associated roads (i.e., MSPA classes: Bridge, Branch, and Loop).

|  | Conservative definition | | | | Broad definition | | | |
| --- | --- | --- | --- | --- | --- | --- | --- | --- |
| Variables | 1986 | 1993 | 2000 | 2009 | 1986 | 1993 | 2000 | 2009 |
| **400-m radius buffer** |  |  |  |  |  |  |  |  |
| Forest (%) | 49.2 ± 39.3 | 48.3 ± 39.3 | 46.2 ± 39.4 | 41.2 ± 39.2 | 49.2 ± 39.3 | 48.0 ± 39.3 | 46.1 ± 39.4 | 41.1 ± 39.2 |
| Exurban development (%) | 1.7 ± 2.5 | 2.1 ± 2.6 | 3.1 ± 3.4 | 6.0 ± 6.8 | 8.6 ± 7.5 | 9.4 ± 7.7 | 10.5 ± 8.3 | 13.8 ±11.1 |
| Forest interior (%) | 39.8 ± 32.2 | 38.1 ± 31.9 | 35.8 ± 31.8 | 29.3 ± 32.4 | 39.8 ± 32.2 | 37.7 ± 32.0 | 35.8 ± 31.8 | 29.3 ± 32.4 |
| Area- weighted average patch size (ha) | 22.2 ± 20.8 | 21.7 ± 20.7 | 20.6 ± 20.6 | 18.5 ± 20.5 | 22.2 ± 20.8 | 21.4 ± 20.7 | 20.6 ± 20.6 | 18.4 ± 20.5 |
| Forest fragments (%) | 23.4 ± 35.7 | 23.5 ± 35.6 | 25.1 ± 37.9 | 31.9 ± 40.9 | 23.4 ± 35.7 | 23.5 ± 35.6 | 25.1 ± 37.9 | 31.9 ± 40.9 |
| Number of forest patches (> 0.45 ha) | 1.7 ± 1.1 | 1.7 ± 1.2 | 1.6 ± 1.2 | 1.6 ± 1.4 | 1.7 ± 1.1 | 1.7 ± 1.2 | 1.6 ± 1.2 | 1.6 ± 1.4 |
| Forest edge (%) | 24.1 ± 14.7 | 24.3 ± 14.8 | 24.5 ± 16.4 | 20.7 ± 16.2 | 24.1 ± 14.7 | 24.1 ± 14.4 | 24.5 ± 16.4 | 20.6 ± 16.2 |
| **1-km radius buffer** |  |  |  |  |  |  |  |  |
| Forest (%) | 51.0 ± 35.7 | 50.0 ± 35.6 | 47.9 ± 35.7 | 42.7 ± 35.8 | 51.0 ± 35.7 | 49.6 ± 35.6 | 47.9 ± 35.7 | 42.7 ± 35.8 |
| Exurban development (%) | 1.8 ± 1.6 | 2.2 ± 1.9 | 3.2 ± 2.6 | 6.2 ± 5.6 | 8.3 ± 5.6 | 9.2 ± 6.0 | 10.2 ± 6.5 | 13.6 ± 9.0 |
| Forest interior (%) | 55.6 ± 28.9 | 53.1 ± 28.9 | 49.4 ± 30.2 | 40.1 ± 32.4 | 55.6 ± 28.9 | 52.2 ± 29.3 | 49.4 ± 30.2 | 40.1 ± 32.4 |
| Area- weighted average patch size (ha) | 134.4 ± 123.5 | 131.8 ± 123.1 | 123.2 ± 121.7 | 111.6 ± 121.3 | 134.4 ± 123.5 | 131.0 ± 122.7 | 123.2 ± 121.7 | 111.6 ± 121.3 |
| Forest fragments (%) | 10.2 ± 17.8 | 11.2 ± 19.6 | 14.4 ± 24.5 | 19.9 ± 28.8 | 10.2 ± 17.8 | 12.0 ± 20.8 | 14.4 ± 24.5 | 19.9 ± 28.8 |
| Number of forest patches (> 0.45 ha) | 5.0 ± 4.2 | 5.0 ± 4.2 | 5.3 ± 4.3 | 5.4 ± 4.4 | 5.0 ± 4.2 | 5.3 ± 4.3 | 5.3 ± 4.3 | 5.4 ± 4.4 |
| Forest edge (%) | 23.6 ± 11.3 | 24.5 ± 11.8 | 24.4 ± 12.6 | 22.5 ± 12.8 | 23.6 ± 11.3 | 24.1 ± 11.9 | 24.4 ± 12.6 | 22.5 ± 12.8 |
